# Cdk5 and Foxc2 – a new relationship in the lymphatic vasculature

**DOI:** 10.18632/oncotarget.4848

**Published:** 2015-07-13

**Authors:** Johanna Liebl

**Affiliations:** ^1^ Ludwig Maximilians-University Munich, Department of Pharmacy, Pharmaceutical Biology, Munich, Germany

**Keywords:** chromosome section, Cdk5, Foxc2, lymphatic vessels, lymphatic valves

## Abstract

Lymphatic vessel dysfunction is associated with various pathologic conditions, including immunologic disorders, lymphedema, as well as tumor dissemination. Yet, the knowledge about the regulation of lymphatic vessel development is still limited. Our study elucidates cyclin dependent kinase 5 (Cdk5) as an essential player in the development of lymphatic vessels. Deletion of Cdk5 in the mouse endothelium results in severe lymphedema formation and embryonic lethality. On the mechanistic level, we show that Cdk5 phosphorylates the forkhead transcription factor Foxc2 which regulates Foxc2-dependent transcription. In summary, our study elucidates the Cdk5-Foxc2 interaction as a critical regulator of lymphatic vessel development.

The lymphatic system is essential for the maintenance of tissue fluid homeostasis, enables the uptake of dietary lipids and regulates the immune response by serving as a trafficking route for immune cells [1]. Lymphatic vessel formation starts after the circulatory system has been established by specification of lymphatic endothelial cells from preexisting embryonic veins. The primary lymph sacs are formed and get separated from the cardinal vein by the formation of lymphovenous valves. Primary lymphatic vessels remodel into blind ending lymphatic capillaries that take up interstitial fluid and collecting lymphatic vessels that contain valves, are covered by smooth muscle cells and drain the lymph into the venous system [2].

Malfunctioning of the lymphatic system is associated with various pathologic conditions. Dysfunction of the lymphatic vasculature results in lymphedema formation and compromises immune function. Moreover, the lymphatic system contributes to tumor cell metastasis – tumors induce lymphangiogenesis to allow the dissemination of tumor cells [3].

The forkhead transcription factor Foxc2 plays a central role in lymphatic vessel development and lymphatic valve formation [4-6]. Mutations in the Foxc2 gene cause lymphedema-distichiasis, a human disease with severe lymphedema and double rows of eyelashes [7, 8]. Moreover, Foxc2 has been associated with cancer cell metastasis and epithelial-mesenchymal transition [9, 10]. Recently, it has been shown that phosphorylation regulates Foxc2-mediated transcription in lymphatic endothelial cells [11]. However, a kinase responsible for Foxc2 phosphorylation has not been identified.

Our recent study elucidates the serine-threonine kinase cyclin dependent kinase 5 (Cdk5) as the missing link in the regulation of Foxc2 in the lymphatic endothelium [12]. The “neuronal kinase” Cdk5 has been supposed to be neuron-specific for a long time due to its essential function in CNS development, function and disease. Although, recently, the awareness about extra-neuronal functions of Cdk5 has grown and Cdk5 was for example associated with cancer, inflammation, or metabolism [13, 14], our knowledge about Cdk5 in the periphery is still insufficient. Only few studies including our own pointed to a function of Cdk5 in the endothelium [15-17] and until now, a detailed study investigating the *in vivo* function of Cdk5 in the endothelium was still missing.

By using endothelial-specific Cdk5 knockout mouse models, our recent work demonstrated that Cdk5 is essential for lymphatic vessel development. Endothelial-specific deletion of Cdk5 causes congenital lymphatic dysfunction with lymphedema and finally results in embryonic lethality. Cdk5 knockdown embryos develop non-separation of blood and lymphatic vessels and show severe defects in lymphatic valve formation and lymphatic vessel patterning with ectopic coverage of lymphatic vessels by smooth muscle cells. As the underlying mechanism, we identified Cdk5 as the upstream kinase of Foxc2. Cdk5 phosphorylates Foxc2 and activates Foxc2-dependent transcription. As a consequence, Cdk5-mediated phosphorylation of Foxc2 regulates the expression of Foxc2-dependent target genes. Among others, connexin 37, a downstream target gene of Foxc2 that is crucial for lymphatic valve formation [6, 18], was strongly diminished in the lymphatic endothelium of Cdk5 deficient mice.

Collectively, our findings elucidate the Cdk5-Foxc2 interaction as a critical regulator of the transcriptional network underlying lymphatic vascular remodeling and thereby provide novel insight into the regulation of lymphatic vessel development. Moreover, because Cdk5 represents a drugable kinase [16, 17], our work implicates Cdk5 as a potential target for the treatment of diseases associated with lymphatic dysfunction.

**Figure:**
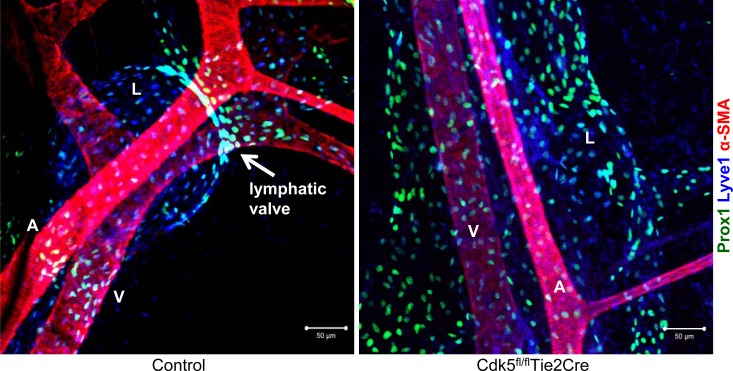
Defective lymphatic valve formation in endothelial-specific Cdk5 knockout mice Whole-mount immunostainings of mesenteric vessels of control and Cdk5 knockout (Cdk5fl/fl/Tie2Cre) embryos (E18.5) are shown. Prox1 (green) and Foxc2 (blue) stain lymphatic endothelial cells, α-smooth muscle actin (α-SMA) stains artery and vein. A lymphatic valve can be recognized by high expression of Prox1 and Foxc2 and is indicated by the arrow in the left picture. A: artery; V: vein; L: lymphatic vessel.
